# Cool Decision-Making in Adolescents with Behavior Disorder and/or Mild-to-Borderline Intellectual Disability

**DOI:** 10.1007/s10802-015-9996-8

**Published:** 2015-03-20

**Authors:** Anika Bexkens, Brenda R. J. Jansen, Maurits W. Van der Molen, Hilde M. Huizenga

**Affiliations:** 1 Department of Psychology, University of Amsterdam, Weesperplein 4, 1018 XA Amsterdam, The Netherlands; 2 Cognitive Science Center Amsterdam, University of Amsterdam, Weesperplein 4, 1018XA Amsterdam, The Netherlands; 3 ’s Heeren Loo Groot Emaus, Groene Allee 46, 3853 JW Ermelo, Netherlands; 4 Department of Psychology, Leiden University, Wassenaarseweg 52, 2333 AK Leiden, The Netherlands; 5 Department of Psychology, Developmental and Educational Psychology, Wassenaarseweg 52, 2333 AK Leiden, The Netherlands

**Keywords:** Decision-making, Behavior disorder, Mild-to-borderline intellectual disability, Decision strategies, Latent group analysis

## Abstract

Adolescents with Behavior Disorders (BD), Mild-to-Borderline Intellectual Disability (MBID), and with both BD and MBID (BD + MBID) are known to take more risks than normal controls. To examine the processes underlying this increased risk-taking, the present study investigated cool decision-making strategies in 479 adolescents (12–18 years, 55.9 % male) from these four groups. Cool decision-making was assessed with the paper-and-pencil Gambling Machine Task. This task, in combination with advanced latent group analysis, allows for an assessment of decision strategies. Results indicated that adolescents with BD and controls were almost equivalent in their decision-making strategies, whereas adolescents with MBID and adolescents with BD + MBID were characterized by suboptimal decision-making strategies, with only minor differences between these two clinical groups. These findings may have important clinical implications, as they suggest that risk taking in adolescents with MBID and in adolescents with BD + MBID can be (partly) attributed to the strategies that these adolescents use to make their decisions. Interventions may therefore focus on an improvement of these strategies.

Although adolescence is a period of positive development such as increasing physical strength and mental capabilities, it is also a period of negative outcomes such as increased mortality and morbidity, which are often attributed to an increase in risk-taking behavior (Crone and Dahl 2012; Steinberg 2008). Within the clinical literature, research on adolescent risk-taking (e.g., Sonuga-Barke and Fairchild 2012) has focused on a group of adolescents that show particularly high levels of risk-taking in daily life, namely adolescents with Behavior Disorders (BD). These adolescents are characterized by persistent, treatment resistant behavioral problems including impulsive, delinquent and oppositional behavior, which can be classified as attention-deficit/hyperactivity disorder, oppositional defiant disorder or conduct disorder (American Psychiatric Association 2013). Adolescents with BD also show a higher incidence of traffic violations (Barkley et al. 1993) and sexual risk-taking (Ramrakha et al. 2007).

These findings are supported by experimental research showing that adolescents with BD make suboptimal decisions on a wide variety of hot decision-making tasks (Fairchild et al. 2009; Garon 2006; Humphreys and Lee 2011; Toplak et al. 2005), that is, on decision-making tasks in which participants have a stake, resulting in a heightened state of emotional arousal when gains or losses are experienced immediately after the decision (e.g., Fairchild et al. 2009; Figner et al. 2009). This suboptimal performance has been attributed to insufficient control over emotions, presumably linked to an altered functioning of the orbitofrontal cortex (Matthys et al. 2012).

An alternative explanation for these findings is that suboptimal decision-making in this population can be attributed to deficits in the cool decision-making process. Cool decision-making is studied in tasks in which decisions are made by weighing and evaluating choice information, without interference from heightened emotional arousal, as decisions in these tasks do not lead to immediate actual consequences. Cool decision-making is omnipresent in daily life and is consequently often studied in the literature (e.g., Jansen et al. 2012; Reyna and Brainerd 2011; Stanovich et al. 2008). The focus in the cool decision-making literature is often on the strategies that are applied to arrive at a decision. These strategies vary in the level of complexity. In simple strategies, only a few dimensions of a decision problem are taken into account which often leads to suboptimal decisions, whereas in more complex strategies more dimensions are considered which often leads to better decisions. The complexity of these strategies is dependent on executive functions such as working-memory and inhibition, that is, individuals high on executive functions are able to apply more complex strategies (DeStefano and LeFevre 2004; Stewart 2009). As there still exists controversy regarding whether adolescents with BD are impaired in their executive functioning (for a review see Matthys et al. 2012), we explore cool decision-making among adolescents with BD. The first aim of this study is therefore to explore whether adolescents with BD are characterized by impaired cool decision-making and, if so, whether this can be explained by their use of suboptimal decision-making strategies.

It should be noted that studies on BD often adopt stringent IQ inclusion criteria, and therefore do not study adolescents with both BD and Mild-to-Borderline Intellectual Disability (MBID). MBID is defined by significant limitations in adaptive skills in addition to an IQ between 50 and 85 (Schalock et al. 2010; Van Nieuwenhuijzen et al. 2011). We argue that it is important to study not only adolescents with BD, but also adolescents with both BD and MBID (BD + MBID) and adolescents with MBID-only. First, it is important to include adolescents with BD and MBID, because this relatively large group (2.5 % of the adolescent population is characterized by BD + MBID; Dekker and Koot 2003; Stoll et al. 2004) places a high burden on both family and society. For instance, an estimated 10 to 40 % of individuals in the criminal justice system, who are often characterized by BD (Fazel et al. 2008), have an IQ below 85 (e.g., see Holland et al. 2002; Kaal 2010; Teeuwen 2012). Second, it is also important to study adolescents with MBID-only in order to disentangle the effects of low IQ and behavior disorder on cool decision-making. In addition, this MBID-only group is of interest in itself as epidemiological studies show that different types of risk behavior (i.e., sexual risk-taking, substance abuse and delinquency) are increased in adolescents with MBID (e.g., Carroll Chapman and Wu 2012; Greenspan et al. 2011).

MBID is characterized by broad deficits in executive functioning (e.g., Bexkens et al. 2013a; Van der Molen et al. 2009), consistent with the finding that indices of fluid intelligence are closely related to those of executive functions (Blair 2006; Stanovich 2009). As executive functions are required for cool decision-making, we hypothesize that adolescents with MBID are characterized by cool decision-making deficits. There is some evidence in support of this hypothesis. Specifically, when making decisions about daily life situations described in vignettes, individuals with intellectual disability show less evidence of evaluation of multiple dimensions of a decision problem (Willner et al. 2010), are characterized by more impulsive decision-making (Jenkinson et al. 1992), and are less able to balance competing goals (Khemka and Hickson 2006). The second aim of the current

study is therefore to replicate the finding that MBID is characterized by impaired cool decision-making, and to test whether this can be explained by suboptimal decision strategies. Finally, the third aim of the current study is to investigate cool decision-making and underlying strategies in adolescents with both BD + MBID. As MBID is characterized by executive function deficits, we test whether adolescents with BD + MBID are characterized by suboptimal cool decision-making as compared to controls. As the literature on executive function deficits in BD is not unequivocal, we explore whether a potential deficit in cool decision-making in adolescents with BD + MBID is more pronounced than in adolescents with MBID-only.

For this purpose, we compared adolescents with BD, with MBID, and adolescents with BD + MBID to typically developing controls. As our aim was not only to assess suboptimal cool decision-making, but also the strategies underlying this decision-making, we administered the Gambling Machine Task (Jansen et al. 2012; Van Duijvenvoorde et al. 2010). This task, in combination with advanced statistical modelling, allows the study of strategies underlying suboptimal decision-making.

## Methods

### Participants

Participants were adolescents between 12 and 18 years of age (55.9 % male, *M*age = 14.6, sd = 1.5). Each adolescent was assigned to one of four groups varying in the presence or absence of BD and MBID. The assignment of adolescents to the clinical groups consisted of two phases.

#### Initial Assignment

The initial assignment was based on school type. Controls were selected from a regular secondary education school. BD students were selected from special education schools for children with a behavior and/or psychiatric disorder. These schools have the following admittance criteria, which are re-evaluated once every 2 years by an independent committee: (1) the student is diagnosed with a DSM-IV (or ICD-10) psychiatric, behavior, or social-emotional disorder by a psychiatrist or clinical psychologist no more than 2 years prior to admittance; (2) the student shows problematic behavior across contexts (e.g., at school and home); (3) student and family support provided by child and adolescent mental health services have not led to remediation of problem behavior; (4) the student has a learning impediment resulting from the diagnosed disorder (e.g., problems with concentration and motivation) or learning delays in at least two areas. These delays cannot be attributed to cognitive ability; and (5) the student’s previous school has undertaken efforts to adapt the school environment, but this has not led to sufficient improvement in the student’s behavior.

BD + MBID students were selected from a special education school for children with behavior and/or psychiatric disorder that specialized in children with low IQ. Admittance criteria were the same as those for BD, with the additional criterion of an IQ between 55 and 85, tested not more than 2 years before admittance.

MBID-only students were selected from special vocational education schools (praktijkonderwijs), focusing on the instruction of practical skills. These schools have the following admittance criteria: (1) an IQ between 55 and 85 tested no more than 2 years prior to admittance; and (2) learning delays of 50 % or more in at least two of the following areas: mathematics, reading accuracy and fluency, reading comprehension, and spelling. One of these delays must be in mathematics or reading comprehension.

#### Refined Assignment

This initial assessment was refined based on the most recent IQ and DSM-IV information from school files. Participants with IQ below 85 were classified in the MBID groups. Participants with IQ of 85 or above were classified in the non-MBID groups. This procedure led to 24 reclassifications from the BD group to the BD + MBID group and 5 reclassifications from the BD + MBID group to the BD group. We only included adolescents in the BD groups if their DSM-IV diagnosis was Oppositional Deviant Disorder (ODD), Conduct Disorder (CD), Disruptive Behavior Disorder NOS, or Attention Deficit/Hyperactivity Disorder (ADHD). Note that all adolescents in the BD and MBID + BD groups, including adolescents with only a diagnosis of ADHD, had persistent, treatment resistant behavioral problems. Note that 10 adolescents were excluded from the MBID-only group because they had additional DSM-IV classifications other than intellectual disability. See Table 1 for participant characteristics in each of the four resulting groups.

**Table 1:** Participant characteristics in each of the four groups

	Control (*N* = 106)	BD (*N* = 103)	MBID (*N* = 152)	BD + MBID (*N* = 118)
Mean age	14.3 (1.4)	14.8 (1.5)	14.5 (1.4)	15.1 (1.8)
Mean IQ	–	97.3 (8.9)	72.4 (6.3)	73.0 (6.9)
% male	51.9	64.1	50.7	61.9
DSM-IV disorders		35 ADHD 62 DBD 6 ADHD + DBD		28 ADHD 72 DBD 18 ADHD + DBD

Standard deviations are reported between parentheses. *BD* behavior disorders, *MBID* mild-to-borderline intellectual disability, *ADHD* attention deficit/hyperactivity disorder, *DBD* disruptive behavior disorder, which includes Oppositional Defiant Disorder and Conduct Disorder

A one-way ANOVA testing the effect of group (Controls, BD, MBID, MBID + BD) on age showed that there was a difference in mean age between groups, *F*(3, 475) = 7.75, *p* < 0.001, *partial η* ^*2*^ = 0.05. Bonferroni corrected post-hoc analyses showed that adolescents in the BD + MBID group were older than adolescents in the MBID (*p* < 0.01) and Control (*p* < 0.01) groups. We therefore included age as a covariate in the analyses on decision-making accuracy. An ANOVA on IQ-scores (from school files) showed that IQ was significantly higher in the BD compared to the MBID (*p* < 0.001) and BD + MBID (*p* < 0.001) groups, whereas IQ did not differ between the latter two groups (*p* = 0.88). IQ scores of children in the control group were not available, however as all children had successfully finished regular primary education we are confident that IQ’s in these adolescents were at least average. As a precaution, teachers were asked to identify children whom they suspected might have lower IQ, but none of the participating adolescents were identified as such. The distribution of gender did not differ significantly between groups, *x* ^2^(3) = 6.82, *p* = 0.08.

Informed consent was obtained from caretakers and adolescents. The study was approved by the ethical committee of the university and complied with relevant laws and guidelines.

### Measures and Procedure

#### Gambling Machine Task (GMT)

**Fig. 1:**
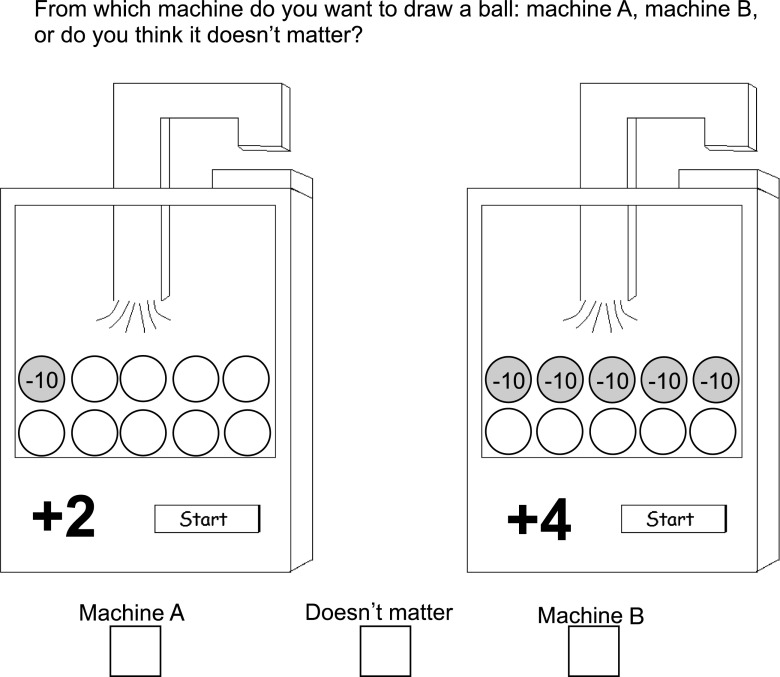
Example item from the GMT. The item shows a conflict between frequency of loss and gain

Cool decision-making was studied with the paper and pencil Gambling Machine Task (GMT, Jansen et al. 2012; Van Duijvenvoorde et al. 2010). This task consisted of several items in which participants had to indicate which of two options was more profitable or whether they thought that both options were equally profitable. The two options, that is, the gambling machines (cf. Fig. 1) were characterized by three dimensions: Gain, loss and frequency of loss. Gain was represented by a positive number on the machine and refers to the number of points the participant gains when that particular machine is chosen. Loss was represented by the negative number in the grey ball(s). Frequency of loss was represented by the number of grey balls in relation to the white balls in the machine.

The task consisted of two practice items and 40 test items (cf. Table 2 for item properties). The task included three simple and seven complex item types, each type was replicated four times. In simple item types, machines differed only on one dimension. In complex item types, machines differed on two or three dimensions and these dimensions led to conflict (e.g., in Fig. 1 machine A has lower frequency of loss, but machine B has higher gain).

**Table 2:** Properties of items from the gambling machine task

	Blocks 1 & 3^a^						Blocks 2 & 4^a^					
	Frequency of loss		Loss		Gain		Frequency of loss		Loss		Gain	
	Machine											
Item Type	A	B	A	B	A	B	A	B	A	B	A	B
fl	10 %	50 %	−2	−2	2	2	50 %	10 %	−50	−50	4	4
l	10 %	10 %	−10	−2	4	4	50 %	50 %	−50	−2	2	2
g	10 %	10 %	−50	−50	4	2	50 %	50 %	−10	−10	4	2
fl_g	10 %	50 %	−10	−10	2	4	50 %	10 %	−50	−50	4	2
l_g	10 %	10 %	−10	−50	2	4	50 %	50 %	−10	−2	4	2
fl_l	50 %	10 %	−2	−10	4	4	50 %	10 %	−10	−50	2	2
g_l	10 %	10 %	−2	−10	2	4	10 %	10 %	−10	−2	4	2
l_fl	10 %	50 %	−50	−2	2	2	10 %	50 %	−50	−2	4	4
l, g_fl	50 %	10 %	−2	−10	4	2	50 %	10 %	−10	−50	4	2
fl_g_l	10 %	50 %	−10	−2	4	2	10 %	50 %	−50	−10	4	2

*fl* frequency of loss, *l* loss, *g* gain. Table adopted from Jansen, et al. (2010)

^a^Items in blocks 1 & 3 are equal, as are items in blocks 2 & 4 except that the left hand and right hand machines were interchanged

The GMT was administered in the adolescents’ classrooms with two experimenters and the teacher present. Although it may be argued that classrooms in both the BD and BD + MBID schools can be more disruptive, these classrooms are also much smaller with only 10 to 15 students. The presence of three adults (i.e., two experimenters and the teacher) ensured that all adolescents were able to finish the task under standardized conditions. Instructions were read aloud by the experimenter. If needed, instructions were repeated. On average, participants needed 15–20 min to finish the GMT.

#### Assessment of Strategy Use

**Fig. 2:**
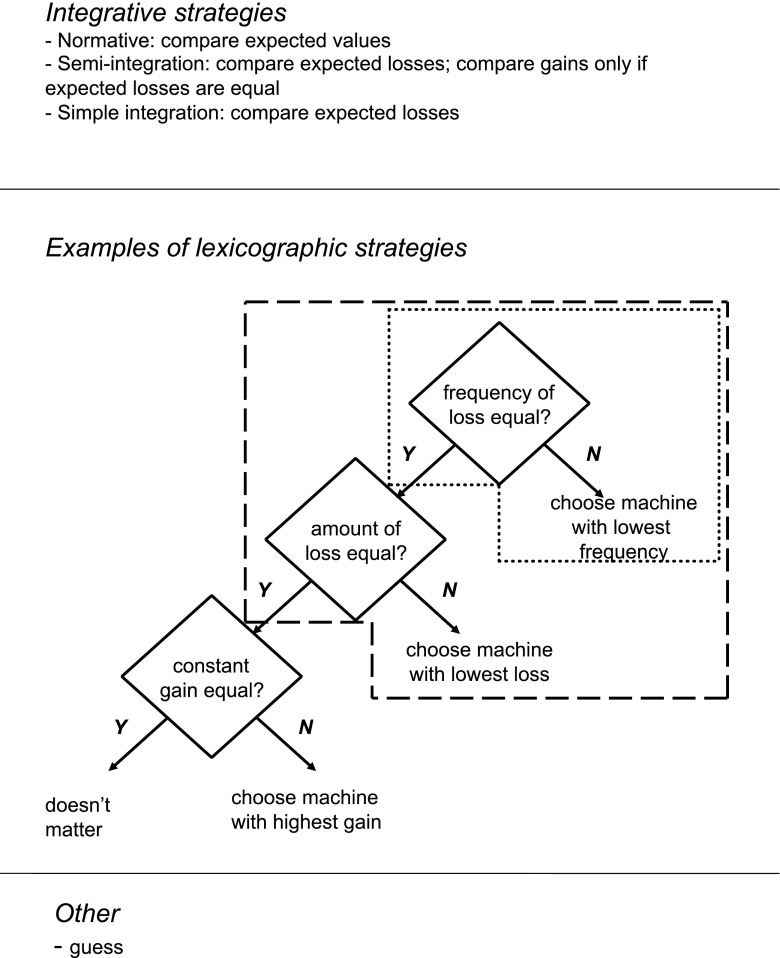
Decision-strategies in assumed order of sophistication. The middle section, entitled ‘Examples of lexicographic strategies’ denotes the decision-making steps in the three-dimensional sequential rule that involves comparing frequency of loss first, amount of loss second, and certain gain third. The section in *dotted lines* denotes the one-dimensional rule that involves considering frequency of loss only. The section in *dashed lines* denotes the two-dimensional rule that involves considering frequency of loss first and amount of loss second

Several strategies can be used to compare the two gambling machines. The normative strategy is to base decisions on expected value, defined by gain plus the probability of loss times the loss itself (normative integration strategy). However, several non-normative strategies exist. For example, another integrative, but non-normative, strategy is to base decisions only on expected losses, that is on the product of probability of loss and the loss, and to only consider gains if expected losses do not differ (semi-integration strategy). An even simpler non-normative integrative strategy is to consider only expected losses (simple integration strategy). Another set of frequently used non-normative strategies are lexicographic strategies (Tversky and Slovic 1988). In lexicographic strategies, dimensions of a decision are considered sequentially. Examples of these lexicographic strategies can be found in Fig. 2, which shows strategies in assumed order of sophistication. The options are first evaluated on one dimension (e.g., probability of loss; see area bordered with dotted lines in Fig. 2). If this dimension discriminates sufficiently between options a decision is made. However, if it does not discriminate sufficiently, a second dimension is evaluated (e.g., loss; see area bordered with dashed lines in Fig. 2). These comparisons may even be followed by comparing the third dimension (e.g., gain). This way, dimensions are evaluated sequentially instead of being integrated (Jansen et al. 2012). An even simpler strategy is to simply guess (guessing strategy). Arguably, integrative strategies are more advanced than sequential strategies, and within sequential strategies, those in which more dimensions are considered to be more advanced than those in which less dimensions are considered. Guessing is considered to be the least advanced strategy.

**Fig. 3:**
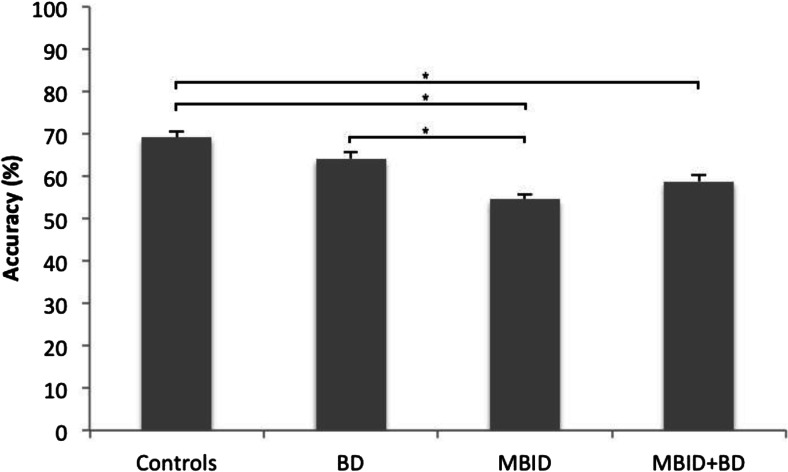
Mean accuracy in percentages in each group. Note: Error bars signify standard errors. *BD* behavior disorders, *MBID* mild-to-borderline intellectual disability. * Signifies a significant difference at a critical alpha value of 0.05. *P*-values were Bonferroni corrected (i.e., multiplied by six)

Previous studies (Jansen et al. 2012; Van Duijvenvoorde et al. 2010) have indicated that the GMT can distinguish these strategies. That is, each of these theoretical strategies elicits a unique GMT response pattern and therefore comparison of observed response patterns to theoretical response patterns offers the possibility to identify underlying strategies. To enhance reliability, we did not compare response patterns of individuals but first grouped individuals into groups with similar response patterns. In order to find these groups, we used latent group analysis (Everitt and Hand 1981), which has been used before in neuroeconomics and in developmental psychology (e.g., Bruhin et al. 2010; Jansen et al. 2012; Van der Maas and Straatemeier 2008; Van Duijvenvoorde et al. 2010). The analysis procedure consisted of three steps (cf. Jansen et al. 2012): (1) we estimated models with 1 to 20 groups, based on the entire data set (i.e., including all control and clinical groups). Each model was run 100 times with different starting values to prevent local minima; (2) we compared the fit (BIC, Leisch 2004) of models with 1 to 20 groups. The model with lowest BIC was selected; (3) in each of the resulting groups, we computed the Euclidean distance between the observed response pattern and each of the theoretical response patterns. The theoretical strategy with lowest Euclidian distance was selected as the strategy that best described observed response patterns.

## Results

### Group Differences in Decision-Making Accuracy

We first analyzed decision-making accuracy, that is, the number of choices that were in accordance with the normative integrative strategy. Decision-making accuracy and IQ were positively correlated over participants, *r(477)* = 0.23, *p* < 0.001. An ANCOVA with Group (Controls, BD, MBID, MBID + BD) as independent variable, Accuracy as dependent variable and Age as covariate showed an effect of Group on accuracy, *F*(3, 474) = 22.08, *p* < 0.001, *partial η* ^*2*^ = 0.12 (cf. Fig. 3). As six contrasts were tested, reported *p*-values were Bonferroni corrected (i.e., multiplied by six). We tested against a critical alpha value of 0.05. These contrasts showed that the performance of adolescents with BD was not different from the performance of to normal controls (*p* = 0.16, *d* = 0.37), whereas the performance of adolescents with MBID and adolescents with BD + MBID was less accurate than normal controls (*p*s < 0.001, *d*s of 1.00 and 0.75 respectively). Adolescents with BD performed better than adolescents with MBID (*p* < 0.001, *d* = 0.61). BD + MBID adolescents tended to perform worse than adolescents with BD-only and tended to perform better than those with MBID-only, although these effects were not significant (*p* = 0.06, *d* = 0.36 and *p* = 0.08, *d* = 0.27 respectively).

### Decision Strategies

Latent group analysis indicated that a model with eight strategy groups described the data best. Response patterns in these strategy groups matched the following strategies (cf. Table 3): guessing, single-dimension, three-dimension (three different strategies), simple integration, semi-integration and normative integration. The three-dimension strategies differed in the order in which dimensions were considered. One of these strategies considered frequency of loss first, followed by gain and loss. A second considered frequency of loss first, then loss and finally gain. A third considered loss first, followed by gain and frequency of loss.

**Table 3:** Response patterns in each decision-strategy group

*n*	Item type										Smallest Euclidian distance to strategy:
	l_g	fl_l	g	fl_g	l	fl,g_l	l_fl	fl	l_g	fl_l, g	
85	0.29	0.45	0.50	0.34	0.35	0.59	0.34	0.32	0.57	0.48	Guessing
	(0.33)	(0.33)	(0.33)	(0.33)	(0.33)	(0.33)	(0.33)	(0.33)	(0.33)	(0.33)	
35	0.47	0.05	0.13	1.00	0.50	0.97	0.10	0.97	0.09	0.02	One dimension (FL)
	(0)	(0)	(0)	(1)	(0)	(1)	(0)	(1)	(0)	(0)	
91	0.44	0.05	0.85	0.94	0.75	0.96	0.08	0.98	0.86	0.09	Three dimensions;(FL, G, L)
	(0)	(0)	(1)	(1)	(1)	(1)	(0)	(1)	(1)	(0)	
73	0.87	0.22	0.87	0.97	0.98	0.85	0.53	0.98	0.24	0.25	Three dimensions;(FL, L, G)
	(1)	(0)	(1)	(1)	(1)	(1)	(0)	(1)	(0)	(0)	
37	0.89	0.10	0.81	0.47	0.90	0.16	0.96	0.53	0.11	0.93	Three dimensions;(L, G, FL)
	(1)	(0)	(1)	(0)	(1)	(0)	(1)	(1)	(0)	(1)	
57	0.83	0.49	0.20	0.90	0.91	0.35	0.82	0.87	0.11	0.21	Simple semi-integration (FL*L)
	(1)	(1)	(0)	(1)	(1)	(0)	(1)	(1)	(0)	(0)	
48	0.92	0.88	0.98	0.96	0.98	0.91	0.97	0.97	0.10	0.87	Semi-integration;((FL*L), G)
	(1)	(1)	(1)	(1)	(1)	(1)	(1)	(1)	(0)	(1)	
66	0.66	0.38	0.93	0.79	0.94	0.74	0.85	0.97	0.68	0.63	Normative integration (G+(FL*L))
	(1)	(1)	(1)	(1)	(1)	(1)	(1)	(1)	(1)	(1)	

*g* gain, *l* loss, *fl* frequency of loss, lowercase refers to item types, uppercase refers to strategies. Response patterns associated to the best fitting strategy are shown in parentheses (cf. main text for their interpretation)

### Group Differences in Decision Strategies

We performed a chi-square test to determine whether there was an association between group (controls, MBID, BD, and BD + MBID) and the eight strategies mentioned in the previous section, which proved to be the case, *x* ^2^(21) = 79.82, *p* < 0.001. In order to determine more precisely how the groups differed in strategy use, we performed pairwise comparisons. *P*-values were Bonferroni corrected for multiple comparisons. All Bonferroni corrected *p*-values were compared to a critical value of 0.05 (cf. Table 4).

**Table 4:** Percentage of individuals within each group using the different strategies

	Control	BD	MBID	BD + MBID	Group Differences
Guess	4.7_a_	11.7_a, b_	29.6_c_	16.1_b, c_	Control < MBID & BD + MBID | BD < MBID
One dimension (FL)	0.0_a_	7.8_b_	9.2_b_	9.3_b_	Control < BD & MBID & BD + MBID
Three dimensions (FL,G,L)	13.2_a_	22.3_a_	17.8_a_	20.3_a_	No group differences
Three dimensions (FL,L,G)	24.5_a_	17.5_a, b_	7.9_b_	14.4_a, b_	Control > MBID
Three dimensions (L,G,FL)	11.3_a_	6.8_a_	7.9_a_	4.2_a_	No group differences
Simple semi-integration (FL*L)	7.5_a_	8.7_a_	13.8_a_	14.4_a_	No group differences
Semi-integration (FL*L,G)	14.2_a_	13.6_a_	3.3_b_	11.9_a_	Control, BD & BD + MBID > MBID
Integrative normative (G + FL*L)	24.5_a_	11._7a, b_	10.5_b_	9.3_b_	Controls > MBID & BD + MBID

Same subscript letters denote no significant differences between groups at α = 0.05 (Bonferroni corrected). *G* gain, *L* loss, *FL* frequency of loss, *BD* behavioral disorders, *MBID* mild to borderline intellectual disability

#### Clinical Groups vs. Controls

Comparisons between BD and controls showed that the single-dimension strategy was more prevalent in BD than controls (cf. Table 4). Prevalence of the other strategies did not differ between BD and controls.

Comparisons between MBID and controls indicated that guessing and the single-dimension strategy were more prevalent in MBID than controls. Conversely, a three-dimension strategy (frequency of loss, loss, gain), the semi-integration strategy and the normative integration strategy were less prevalent in MBID than in controls. Prevalence of other strategies did not differ between MBID and controls.

Comparisons between BD + MBID and controls revealed that guessing and the single-dimension strategy were more prevalent in BD + MBID than controls. On the other hand, the normative integration strategy was less prevalent in BD + MBID than controls. Prevalence of other strategies did not differ between BD + MBID and controls.

In sum, the less advanced decision strategies (i.e., the guessing and the single dimension strategy) were more prevalent in clinical groups as compared to controls, whereas more advanced strategies (i.e., the three dimension and integrative strategies) were less prevalent in MBID and BD + MBID groups as compared to controls.

#### Comparisons Between Clinical Groups

The BD and BD + MBID groups did not differ in the prevalence of any of the strategies. A comparison of the MBID and BD + MBID groups revealed that the semi-integration strategy was less prevalent in the MBID group than the BD + MBID group. There were no differences in the prevalence of other strategies. These results suggest that the decision-making deficit is not more pronounced in adolescents with BD + MBID than in adolescents with either BD or MBID-only. More specifically, the prevalence of strategies did not differ between BD and BD + MBID groups and decision-making strategies were less, instead of more, advanced in adolescents with MBID as compared to those with BD + MBID.

Comparisons between BD and MBID groups showed that guessing was more prevalent in MBID than in BD. In addition, the semi-integration strategy was less prevalent in MBID than BD. Taken together these results show that decision strategies were less advanced in MBID than in BD.

### Additional Checks on Age, Gender, Medication Use, Disorder Type, and Level of Intellectual Disability

There was a small negative correlation between decision accuracy and age, indicating lower accuracy in older participants, *r*(477) = −0.10, *p* = 0.03. Decision accuracy was not associated with gender, *F*(1, 477) = 0.85, *p* = 0.36, *partial η* ^*2*^ = 0.002. Neither was decision accuracy related to medication use,^1^ *F*(1, 219) = 0.54, *p* = 0.46, *partial η* ^*2*^ = 0.002 or disorder type (ADHD vs ODD/CD vs ADHD + ODD/CD) within adolescents with BD (BD-only and BD + MBID), *F*(2, 218) = 0.53, *p* = 0.59, *partial η* ^*2*^ = 0.005.

Strategy use was not associated with age, *F*(7, 471) = 1.87, *p* = 0.07, *partial η* ^*2*^ = 0.03, gender, *x* ^*2*^(7) = 6.68, *p* = 0.46, medication use, *x* ^*2*^(7) = 6.63, *p* = 0.47, or disorder type (ADHD vs ODD/CD^2^), *x* ^*2*^(7) = 8.14, *p* = 0.32.

In contrast to previous studies, only a small group of MBID and BD + MBID participants used a single-dimension strategy. A possible explanation is that previous studies of decision-making included participants with Mild Intellectual Disability (MID, IQ between 50 and 70), whereas we also included participants with Borderline Intellectual Disability (BID, IQ between 70 and 85). To test this explanation, MBID and BD + MBID participants were divided in MID and BID groups, which were compared on strategy use. There was no significant association between level of intellectual disability and strategy use in the group without BD, *x* ^2^(7) = 9.90, *p* = 0.17 nor the group with BD, *x* ^2^(7) = 12.52, *p* = 0.09.

### Additional Check on Task Understanding

Considering that adolescents with MBID have low IQ, it is important to check whether they were sufficiently able to perform the task. All adolescents completed the 40 choice items, however this does not necessarily indicate that they understood the task (cf. Willner et al. 2010). One of the strengths of latent class analysis, however, is that it allows for identification of decision-strategies, amongst which guessing. This analysis indicated that guessing was more prevalent in both the MBID-only and the MBID + BD groups than in normal control group, which might indicate that a subset of adolescents were not able to formulate a consistent decision strategy, although the majority of adolescents within each group was able to do so.

## Discussion

Risky decision-making by adolescents with Behavior Disorders (BD) is often attributed to insufficient control over emotion. However, an alternative explanation is that the cool decision-making process itself is impaired. In the current study, we investigated such cool decision-making by adolescents with Behavior Disorders (BD), Mild-to-Borderline Intellectual Disability (MBID) and adolescents with both BD and MBID, by comparing them to normal controls. We did this by administering a task, the Gambling Machine Task (Jansen et al. 2012). This task allows the assessment of cool decision-making accuracy, indexed by the number of responses in accordance with the normative integrative strategy. More importantly, it allows the assessment of strategies underlying decisions, thereby providing more insight into the origins of suboptimal cool decision-making.

Adolescents with BD did not differ from controls in their decision-making accuracy. In addition, strategy analysis indicated that only the less advanced single-dimension strategy was more prevalent in the BD group than in the control group, whereas there were no further strategy differences between BD and controls. The current observation may suggest that most adolescents with BD do not experience decision-making deficits in cold contexts. The implications of this finding are twofold. First, we argue that it is likely that their difficulties in decision-making only become apparent in hot contexts in which outcomes are experienced immediately (e.g., Rubia 2011), consistent with a deficit in control over emotion (Matthys et al. 2012). Second, as cool decision-making relies on executive functions such as working memory and inhibition (DeStefano and LeFevre 2004; Stewart 2009), the observation that cool decision-making is not impaired in BD, may be taken as additional evidence for the notion that executive functions are intact in adolescents with BD (cf. Oosterlaan et al. 2005).

Adolescents with MBID were, as compared to controls, characterized by lower decision-making accuracy and the underlying strategies were less advanced. This finding is consistent with previous studies of cool decision-making in adults with MBID (Jenkinson et al. 1992; Khemka and Hickson 2006; Willner et al. 2010). Given the association between cool decision-making and executive functions, this result also provides further evidence that the MBID population is characterized by severe deficits in executive functioning.

Adolescents with BD + MBID were, as compared to controls, characterized by lower decision-making accuracy, moreover decision strategies were less advanced in this group. Therefore we conclude that adolescents with BD + MBID are impaired in their cool decision-making. Adolescents with BD + MBID did not differ from adolescents with BD in accuracy or in decision strategies. In addition, adolescents with BD + MBID did not differ from adolescents with MBID in accuracy, and strategy analysis indicated somewhat better, instead of worse, performance in the BD + MBID group.

The finding that adolescents with BD + MBID performed slightly better than those with MBID was unexpected. One potential explanation for this finding is that the BD + MBID group was slightly older. However, as we did not find an association between age and strategy use, this explanation is unlikely. A second potential explanation is that executive function deficits are less pronounced in BD + MBID than in MBID. Although this might seem implausible, there is evidence to suggest that executive functions are indeed less impaired in adolescents with BD + MBID than in MBID (Bexkens et al. 2013b; Ponsioen and Van der Molen 2002). It has been argued that in adolescents with BD, a low IQ, and thus the diagnosis MBID, may be secondary to the behavior disorder, whereas low IQ might be a primary characteristic in adolescents with only MBID (Bexkens et al. 2013b). For example, behavior disorder is associated with classroom underachievement (Pardini and Fite 2010; Timmermans, Van Lier, and Koot 2010) and thus may also lead to underachievement on IQ tests. In sum, the present results, together with previous findings very tentatively suggest that MBID in adolescents with or without BD is of a qualitatively different nature (cf. Ponsioen and Van der Molen 2002), and therefore it might be better to treat BD + MBID as a separate diagnostic category instead of as the sum of two diagnoses. However, further research is clearly required to test this hypothesis.

The current study has several implications. First, the finding that only adolescents with MBID-only and adolescents with BD + MBID experience decision-making deficits in cold contexts has important implications for interventions targeting risky behavior. Although further research is needed to replicate this finding, it suggests that for adolescents with BD, interventions need not to focus on the cool decision-process itself. For adolescents with MBID and adolescents with BD + MBID, however, interventions should target the cool decision-making process and interventions focused on weighing and evaluating information may be effective. Given the large individual differences in quality of decision-making strategies within groups, another fruitful approach may be to study the source of these individual differences, as this may provide insight into which abilities to target in intervention programs.

Second, previous studies indicate that individuals with mild intellectual disabilities (IQ range 50–70) considered only one dimension in their decision-making (Willner et al. 2010). Adolescents with MBID in our study clearly used more advanced strategies, as they were able to consider three dimensions and could even make simple integrations between two dimensions. This apparent discrepancy cannot be explained by the inclusion of adolescents with Borderline Intellectual Disability in our study, as our analysis indicated no association between strategy use and level of intellectual disability. The task used by Willner and colleagues (2010) might have been more difficult than the GMT, because the verbally presented vignettes require efficient use of working memory capacity to remember and evaluate all choice options. Therefore, we tentatively conclude that visual presentation may help adolescents with MBID to reach better decisions (cf. Bailey et al. 2011).

Third, a substantial subgroup of adolescents with MBID and BD + MBID used quite advanced decision-strategies. Future studies should focus on the source of these individual differences in cool decision-making within the MBID group. A likely origin of these individual differences in cool decision-making is executive functioning ability, which may vary even within the group diagnosed with MBID (Willner et al. 2010).

Fourth, although the primary focus of this study was to compare distinct groups, we also checked whether there was a relation between continuous IQ and decision-making accuracy in the entire sample, which proved to be the case. In contrast, studies using university populations show very little evidence of an association between decision-making accuracy and cognitive ability (Stanovich and West 2008). Follow-up studies are needed to test the association between IQ and cool decision-making accuracy in the normal population across a substantial IQ range.

In conclusion, we found that cool decision-making is intact in adolescents with BD, suggesting that their risky decisions in hot contexts should not be attributed to deficient cool decision-making, but is more likely to be related to insufficient control over emotion.

Adolescents with BD + MBID and adolescents with MBID-only however, do experience cool decision-making deficits, suggesting that risky decisions in this subgroup can partly be attributed to deficits in the cool decision-making process itself. This has important implications for interventions to reduce risk-taking behavior in these clinical groups.

## Footnotes

1 Twenty adolescents in the BD and twenty-six adolescents in the BD + MBID group used medication at time of testing. Medication included methylphenidate, atomoxetine and risperidone.

2 Because the ADHD + ODD/CD group was quite small and this resulted in too many nearly empty cells, we excluded this group from this analysis
